# Voluntary Medical Male Circumcision: A Framework Analysis of Policy and Program Implementation in Eastern and Southern Africa

**DOI:** 10.1371/journal.pmed.1001133

**Published:** 2011-11-29

**Authors:** Kim E. Dickson, Nhan T. Tran, Julia L. Samuelson, Emmanuel Njeuhmeli, Peter Cherutich, Bruce Dick, Tim Farley, Caroline Ryan, Catherine A. Hankins

**Affiliations:** 1 World Health Organization, Geneva, Switzerland; 2 Johns Hopkins Bloomberg School of Public Health, Baltimore, Maryland, United States of America; 3 United States Agency for International Development, Washington, District of Columbia, United States of America; 4 Ministry of Public Health and Sanitation, Nairobi, Kenya; 5 Office of the U.S. Global AIDS Coordinator, United States Department of State, Washington, District of Columbia, United States of America; 6 Joint United Nations Programme on HIV/AIDS, Geneva, Switzerland; Centers for Disease Control and Prevention, United States of America

## Abstract

**Background:**

Following confirmation of the effectiveness of voluntary medical male circumcision (VMMC) for HIV prevention, the World Health Organization and the Joint United Nations Programme on HIV/AIDS issued recommendations in 2007. Less than 5 y later, priority countries are at different stages of program scale-up. This paper analyzes the progress towards the scale-up of VMMC programs. It analyzes the adoption of VMMC as an additional HIV prevention strategy and explores the factors may have expedited or hindered the adoption of policies and initial program implementation in priority countries to date.

**Methods and Findings:**

VMMCs performed in priority countries between 2008 and 2010 were recorded and used to classify countries into five adopter categories according to the Diffusion of Innovations framework. The main predictors of VMMC program adoption were determined and factors influencing subsequent scale-up explored. By the end of 2010, over 550,000 VMMCs had been performed, representing approximately 3% of the target coverage level in priority countries. The “early adopter” countries developed national VMMC policies and initiated VMMC program implementation soon after the release of the WHO recommendations. However, based on modeling using the Decision Makers' Program Planning Tool (DMPPT), only Kenya appears to be on track towards achievement of the DMPPT-estimated 80% coverage goal by 2015, having already achieved 61.5% of the DMPPT target. None of the other countries appear to be on track to achieve their targets. Potential predicators of early adoption of male circumcision programs include having a VMMC focal person, establishing a national policy, having an operational strategy, and the establishment of a pilot program.

**Conclusions:**

Early adoption of VMMC policies did not necessarily result in rapid program scale-up. A key lesson is the importance of not only being ready to adopt a new intervention but also ensuring that factors critical to supporting and accelerating scale-up are incorporated into the program. The most successful program had country ownership and sustained leadership to translate research into a national policy and program.

*Please see later in the article for the Editors' Summary*

## Abstract 2

Kim Dickson and colleagues analyze the progress made by 13 priority countries toward scale-up of medical male circumcision programs, finding that the most successful programs involve country ownership of the program and have sustained leadership at all levels.

## Editors' Summary

**Background:**

Every year, more than 2.5 million people (mostly in sub-Saharan Africa) become infected with HIV, the virus that causes AIDS. There is no cure for HIV/AIDS and no HIV vaccine. Consequently, global efforts to combat HIV/AIDS are concentrating on evidence-based prevention strategies such as voluntary medical male circumcision (VMMC). Circumcision—the removal of the foreskin, a loose fold of skin that covers the head of the penis—reduced HIV transmission through sexual intercourse by 60% in men in trials undertaken in sub-Saharan Africa, so in 2007, the World Health Organization (WHO) and the Joint United Nations Programme on HIV/AIDS (UNAIDS) recommended implementation of VMMC programs in countries with a generalized HIV epidemic and low levels of male circumcision. They also identified 13 countries in southern and eastern Africa as high priority countries for rapid VMMC scale-up. Mathematical modeling suggests that 20.3 million circumcisions by 2015 and 8.4 million circumcisions between 2016 and 2025 are needed to reach 80% VMMC coverage in these countries. If this coverage is achieved, it will avert about 3.4 million new HIV infections through 2025.

**Why Was This Study Done?:**

Despite convincing evidence that VMMC is an effective, cost-saving intervention in the fight against HIV/AIDS, national VMMC scale-up programs in the priority countries are currently at very different stages. A better understanding of the challenges faced by these programs would help countries still in the early stages of VMMC scale-up implement their national programs and would facilitate implementation of other HIV prevention strategies. In this study, the researchers use the Diffusion of Innovations (DOI) theory to analyze progress towards VMMC scale-up in the priority countries and to identify the factors that may have expedited or hindered program scale-up. This theory seeks to explain how, why, and at what rate new ideas and technology spread through cultures. It posits that a few individuals (“innovators”) adopt new ideas before they become mainstream ideas. A few more individuals—the “early adopters”—follow the innovators. The “early majority” is the next group to adopt the innovation, followed by the “late majority” and the “laggards.”

**What Did the Researchers Do and Find?:**

The researchers used the annual number of VMMCs performed in the priority countries since 2008 to classify the countries into DOI adopter categories. They calculated a total scale-up score for each country based on six key elements of program scale-up (such as whether and when a VMMC policy had been approved). Finally, they analyzed the association between the DOI adopter category and the scores for the individual scale-up elements to determine which elements predict adoption and VMMC scale-up. By the end of 2010, about 560,000 VMMCs had been completed, less than 3% of the target coverage for the priority countries. Kenya, the only DOI innovator country, had completed nearly two-thirds of the VMMCs needed to reach its target coverage and was the only country on track to reach its target. The early adopters (South Africa, Zambia, and Swaziland) had initiated VMMC program scale-up soon after the release of the 2007 recommendations and had started VMMC scale-up pilot programs in 2008 but were far from achieving their VMMC targets. Having a VMMC focal person, establishing a national policy, having an operational strategy, and establishing a pilot program all predicted early adoption of VMMC scale-up.

**What Do These Findings Mean?:**

These findings show that, three years after the WHO/UNAIDS recommendation to integrate VMMC into comprehensive HIV prevention programs, VMMC scale-up activities had been initiated in all the priority countries but that progress towards the 80% coverage target was variable and generally poor. Importantly, they show that early adoption of VMMC as a national program had not necessarily resulted in rapid program scale-up. Although these findings may not be generalizable to other settings, they suggest that countries endeavoring to scale up VMMC (or other HIV prevention strategies) must not only be ready to adopt VMMC but must also ensure that all the factors critical to supporting and accelerating scale-up are incorporated into the scale-up program. Finally, these findings show that the most successful national programs are those that involve country ownership of the program and that have sustained leadership at all levels to facilitate the translation of research into national policies and programs.

**Additional Information:**

Please access these websites via the online version of this summary at http://dx.doi.org/10.1371/journal.pmed.1001133.

- This study is part of a PLoS Collection of articles on VMMC  (http://www.ploscollections.org/VMMC2011) and is further discussed in a *PLoS Medicine* Review Article by Hankins et al.  (http://dx.doi.org/10.1371/journal.pmed.1001127)
- Information is available from WHO, UNAIDS, and PEPFAR on all aspects of HIV/AIDS
- NAM/aidsmap provides basic information about HIV/AIDS, summaries of recent research findings on HIV care and treatment, and information on male circumcision for the prevention of HIV transmission
- Information is available from Avert, an international AIDS charity on many aspects of HIV/AIDS, including information on aspects of HIV prevention, and on HIV/AIDS in Africa (in English and Spanish)
- The Clearinghouse on Male Circumcision, a resource provided by WHO, UNAIDS, and other international bodies, provides information and tools for VMMC policy development and program implementation
- Wikipedia has a page on Diffusion of Innovations theory (note: Wikipedia is a free online encyclopedia that anyone can edit; available in several languages)
- Personal stories about living with HIV/AIDS are available through Avert, through NAM/aidsmap, and through the charity website Healthtalkonline

## Introduction

In 2009, more than 25 y after HIV was first identified, 2.6 million people had become infected, and there were an estimated 33.3 million people living with HIV worldwide [1]. In the absence of a vaccine, the next best means to combat new HIV infections is the implementation of evidence-based prevention strategies including male and female condoms [2], antiretroviral prophylaxis to prevent vertical transmission from mother to child [3],[4], harm reduction for people who inject drugs [5], and, most recently, treating HIV-infected people in serodiscordant couples with antiretroviral drugs to reduce transmission to partners [6]. However, the scale-up of these HIV prevention programs remains challenging despite evidence demonstrating their effectiveness. The use of male and female condoms, despite decades of promotion and distribution, remains suboptimal. The use of female condoms as a prevention strategy for HIV is reportedly lower than male condom use, including in countries in sub-Saharan Africa, where the majority of new HIV infections are occurring through heterosexual transmission [7] and are predominantly among women.

Evidence demonstrating the effectiveness of voluntary medical male circumcision (VMMC) in preventing HIV sexual transmission was first released in 2005 from the South Africa (Orange Farm) randomized controlled trial (RCT) [8]. This was followed by results in 2006 from RCTs in Uganda (Rakai District) and Kenya (Kisumu) [9],[10]. All three RCTs confirmed that male circumcision performed by well-trained and equipped medical providers is safe and reduces the risk of heterosexual acquisition of HIV infection among men by as much as 60%. These RCT results confirmed decades of evidence from observational studies suggesting male circumcision's strong protective effect for men against HIV [11]. Male circumcision also has a strong protective effect against other sexually transmitted infections in men and in women [12]–[16]. Although there is no conclusive evidence that medical male circumcision has a direct effect on women's risk of HIV infection [17], a systematic review, largely based on observational studies, estimated an overall 20% lower HIV incidence in female partners of circumcised men, compared with partners of uncircumcised men [18].

Following the release of the results of the RCTs, in 2007 the World Health Organization (WHO) and the Joint United Nations Programme on HIV/AIDS (UNAIDS) convened an international consultation of stakeholders from a range of disciplines to review the body of evidence from the three trials and the wealth of earlier ecological and observational studies [19]. The consultation resulted in a firm endorsement of the evidence from the three trials, and the formulation of eleven key conclusions and recommendations for the implementation and scale-up of VMMC programs in countries and settings with generalized high-prevalence HIV epidemics and low levels of male circumcision [19].

WHO and UNAIDS identified 13 countries in southern and eastern African as high-priority countries for the implementation and rapid scale-up of VMMC programs: Botswana, Kenya, Lesotho, Malawi, Mozambique, Namibia, Rwanda, South Africa, Swaziland, Tanzania, Uganda, Zambia, and Zimbabwe. These countries have been working towards the implementation of VMMC programs using operational guidance developed by WHO and UNAIDS. The guidance emphasizes ten essential components of program implementation: leadership and partnerships, situation analysis, advocacy, enabling policy and regulatory environments, national strategy and operational plans, quality assurance and improvement, human resource development, commodity security, social change communication, and monitoring and evaluation [20].

A consensus report of six different models showed that in settings with high HIV prevalence and low circumcision prevalence, one new HIV infection could be averted for every five to 15 circumcisions performed [21]. More detailed modeling was incorporated into the Decision Maker's Program Planning Tool (DMPPT), and it is estimated that scaling up circumcision programs to reach 80% of adult uncircumcised men within 5 y in 13 priority countries would require a total of 20.3 million circumcisions to be performed and a further 8.4 million between 2016 and 2025, averting an estimated 3.4 million new HIV infections and 386,000 AIDS deaths through 2025 [22]. Despite convincing results demonstrating that VMMC is an effective, cost-saving intervention in the fight against HIV, there have been major challenges and barriers to implementing programs in the high priority countries.

This paper analyzes the progress towards achievement of VMMC for HIV prevention program scale-up in 13 priority countries. We analyze the adoption of VMMC as an additional HIV prevention strategy. We further explore the factors that may have expedited or hindered the adoption of VMMC policies and strategies as well as initial program implementation in the 13 priority countries to date. We also explore the factors that may have influenced subsequent program scale-up. Challenges encountered and lessons learned are highlighted for application to countries still in the early stages of VMMC scale-up as well as to other potential HIV prevention strategies such as topical (vaginal and rectal microbicides) and oral pre-exposure prophylaxis.

## Methods

The actual numbers of VMMCs performed in priority countries per calendar year since 2008 were recorded and the totals used to classify countries into five adopter categories according to the Diffusion of Innovations (DOI) framework. Country progress towards achievement of the goal of circumcising 80% of eligible men was also calculated [22].

**Figure 1:**
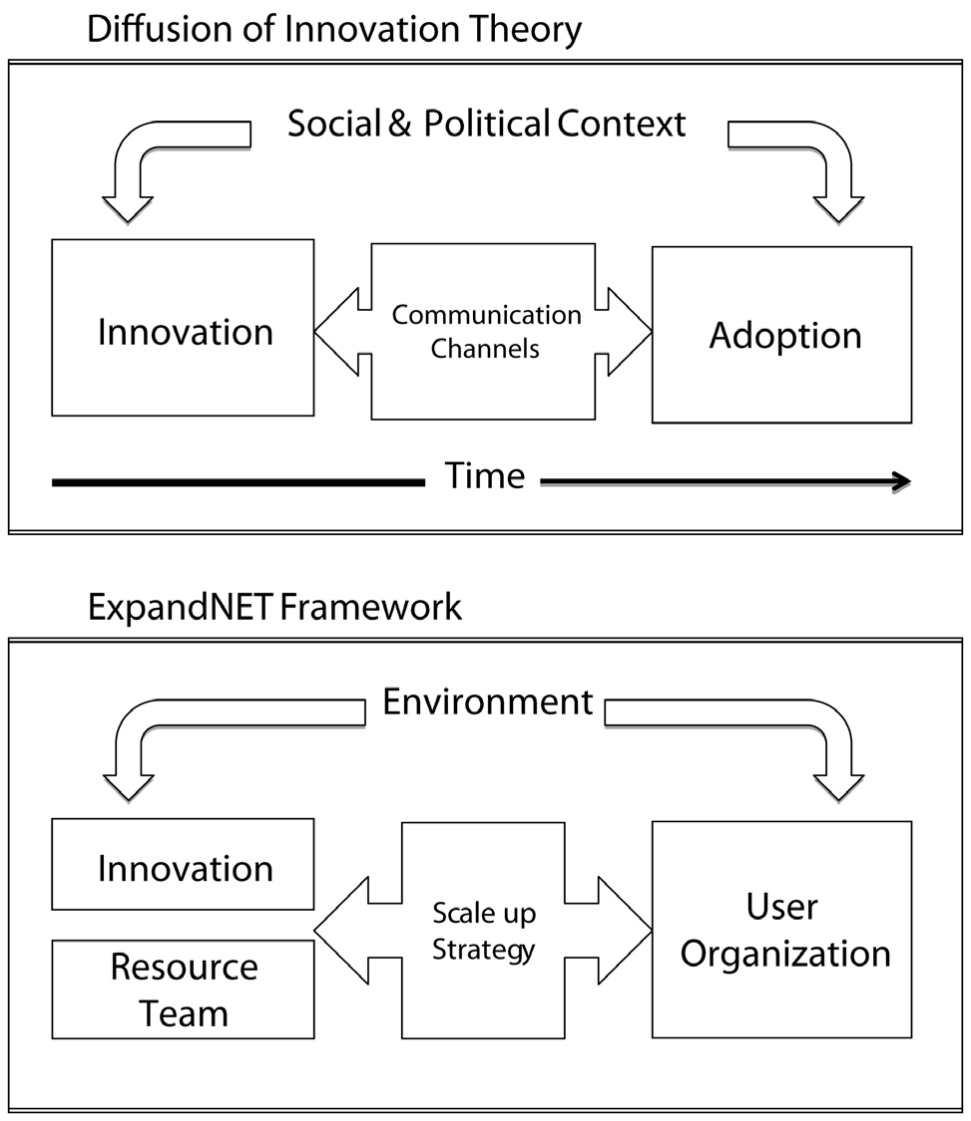
Comparison of Diffusion of Innovation and ExpandNet frameworks. A comparison of the DOI and ExpandNet frameworks is shown. Whereas the DOI describes the process through which innovations are adopted and diffused through the population, the ExpandNet framework specifically addresses the diffusion and scale-up of public health interventions. As highlighted in the figure, the ExpandNet framework reflects the earlier thinking of the DOI and incorporates the elements of context as environment, communication channels as scale-up strategy, and adoption by the population as adoption by the organization implementing the intervention.

The DOI and ExpandNet frameworks were selected to analyze the status of VMMC programming in the 13 priority countries as they are complimentary theories that both refer to the process by which innovations are disseminated and taken to scale. The DOI and ExpandNet frameworks highlight the importance of the nature of the innovation itself and the sociopolitical context or environment in which diffusion takes place (see Figure 1). Whereas the DOI theory addresses the diffusion of any innovation, the WHO ExpandNet framework explicitly addresses the adoption and scale-up of public health programs and services in the health sector. Both frameworks stress that innovations that are **C**redible, **O**bservable, and **R**elevant; have **R**elative advantages; are **E**asy to install and understand; and are **C**ompatible and **T**estable (“CORRECT”) are most likely to be successfully adopted and scaled up [23].

**Figure 2:**
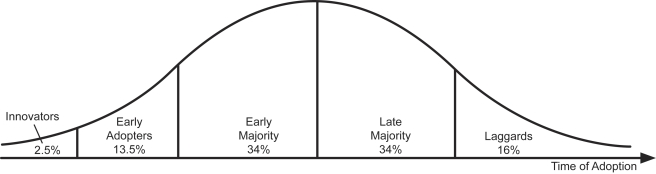
Diffusion of Innovation adoption status. The typical distribution of categories of adopters according to the DOI theory is shown in this figure. The very first adopters or innovators typically represent 2.5% of the population. The next group of adopters, called early adopters, represent about 13.5% of the population and are followed by the early majority, who represent approximately 34% of the total population. The next 34% of the population to adopt are the late majority; this group is followed by the remaining 16% of the population, who are the laggards, the last to adopt a new innovation.

The DOI theory defines diffusion as the process by which an innovation is communicated through certain channels over time among members of a social system [24]. The DOI theory posits that regardless of the setting, initially there are a few individuals who adopt innovations, known as the “innovators”—these are those who are willing to adopt new ideas before they are mainstream. Following this, another small proportion, the “early adopters,” of the population follow. The “early majority” is the next group to adopt the innovation. By this time, more than half of the population has adopted the innovation. The “late majority” is the next group to accept new interventions; the “laggards” are the last to adopt innovations, they represent those who are least likely to accept innovation (see Figure 2). While the DOI theory refers to the adoption pattern of individuals within a community, we applied these concepts to analyze the adoption of VMMC policies, strategies, and initial program implementation by countries. The DOI framework was used to define the adoption status of each country to comparatively assess progress towards scale-up among the 13 priority countries.

In order to facilitate analysis using the DOI framework, six program components based on key elements defined as essential for the operationalization of VMMC programs described in the WHO/UNAIDS operational guidance were used to assess overall country progress towards VMMC program scale-up [20]. The six elements selected were those for which progress towards scale up over a 3-y period could be objectively quantified. A country was assigned a score for each key element, ranging from 0 (no progress) to 3 (maximum progress). A total scale-up score, a composite indicator of progress in program scale-up, was calculated for each country. Additionally, further analyses were undertaken in order to understand if there were differences in the contributions of the six key elements to the overall DOI adoption status, since one can argue that some elements are better predictors of adoption and scale-up than others. To do this, the association between the DOI classification “adopter status” and the scores on the individual elements of scale-up was determined using a crude estimation of association based on an R-squared analysis.

The ExpandNet framework is based on the theory underpinning the DOI model and supplemented with experiences applying the model in low-resource settings [25]. The ExpandNet framework views scaling up as an open system that draws on five interacting elements: the innovation itself, the resource team, the scale-up strategy, the user organization, and the environment. We used the ExpandNet framework to explore the factors that may have influenced subsequent program scale-up, highlighting factors specific to VMMC.

## Results

The “CORRECT” attributes of VMMC are highlighted in Table 1. Male circumcision, an old procedure but an innovation for HIV prevention, has many of the “CORRECT” attributes needed to enhance scalability. VMMC is **C**redible, with consistent evidence of reduced HIV rates from numerous **O**bservational studies and RCTs conducted by respected researchers in Africa, where the intervention is **R**elevant, as it provides a new solution to address a high-burden public health problem; VMMC has **R**elative advantages, as it is a one-time intervention conferring lifetime reduction in risk of HIV infection. Medical male circumcision is not particularly **E**asy to install, as it is a surgical intervention that requires training and expertise, with complex social and cultural barriers that need to be overcome as programs are scaled up. However, it is a one-time intervention, provides a rare opportunity to reach men, and it is cost saving [22],[26]. Medical male circumcision is **C**ompatible with existing national priorities for HIV prevention in the priority countries. In all priority countries, VMMC services have been or are being pilot **T**ested, providing critical information for moving to scale.

**Table 1:** ExpandNet innovation characteristics.

Innovation Element	Key Questions for Scale-Up	Male Circumcision
Credible	1. Have results of pilot testing the innovation been documented? 2. How sound is the evidence? 3. Is further evidence/better documentation needed? 4. Has the innovation been tested in the type of setting where it will be scaled up?	Ecological studies show lower prevalence of HIV infection in countries with high rates of circumcision.
		Epidemiological studies show that circumcised men have a consistently lower incidence of HIV than uncircumcised men, even after adjusting for differences in sexual behavior.
		Three independent RCTs showed that circumcision reduced the risk of HIV infection in young men.
		Research was conducted by credible researchers in directly relevant settings in African countries.
		Male circumcision is not a new procedure, but is an innovation for HIV prevention, with potential for significant impact in countries with generalized HIV epidemics and low prevalence of circumcision.
Observable	How observable are results?	Results from the epidemiological studies and RCTs are unequivocal in demonstrating lower HIV incidence in circumcised men.
		Impact of program scale-up on incidence of HIV infection will take some time to be realized.
		Impact on AIDS and AIDS-related mortality will be even more distal.
		Modeling, costing, and impact studies indicate that VMMC is cost saving and will benefit both men and women.
Relevant	Does the innovation address a felt need, persistent problem, or policy priority?	VMMC addresses the persistent problem of finding ways to prevent HIV in generalized heterosexual epidemics.
		It is directly relevant in southern and eastern African countries that have the greatest HIV incidence and burden of infection.
Relative advantages	1. Does the innovation have relative advantage over existing practices? 2. Is it more cost-effective than existing practices or alternatives?	VMMC is only a one-time intervention, resulting in lifelong lower risk of HIV infection in men.
		VMMC programs are a rare opportunity to reach young men through health services and provide good sexual and reproductive health and HIV risk reduction counseling.
		VMMC has been demonstrated to be highly cost-effective and cost saving for all priority countries. The potential impact is substantially greater than other HIV prevention interventions.
Ease to install	1. What degree of change from current norms, practices, and levels of resources is implied in the innovation? 2. What is the level of technical sophistication needed to introduce the innovation? 3. Are major additional human or financial resources and commodities needed to introduce the innovation?	VMMC is a challenging intervention to implement since it requires surgical skills that are in short supply in the Africa region.
		The number of circumcisions necessary to achieve rapid impact on the HIV epidemic is large, with consequently potentially large implications for human resources, facilities, and supplies.
		VMMC is a straightforward minor outpatient surgical procedure, but must be performed by adequately trained and equipped teams.
Compatible	1. Is the innovation compatible with current values or services of the user organization? 2. Will it be difficult to maintain the basic values of the innovation as expansion proceeds? 3. Will changes in logistics need to be made to accommodate the innovation? 4. Which components will need local adaptation to be relevant for changes in local context?	VMMC is consistent with already existing national priorities for comprehensive HIV prevention.
		There are a wide range of sociocultural factors that need to be considered when scaling up VMMC programs.
		Countries need to ensure that VMMC is promoted in a culturally sensitive way and does not introduce stigma associated with circumcision status.
		The implications of VMMC for women also need to be taken into account when scaling up programs.
Testable	Can the user organization test the innovation in stages without fully adopting it?	Pilot projects have been set up in all 13 priority countries and tailored to local contexts. The pilots have provided information for subsequent program scale-up.
		Since VMMC scale-up requires substantial infrastructure and human and financial resources, incremental approaches to scale-up have been used.
		Best combination for service delivery scale-up is yet to be determined, as well as how to balance supply and demand creation.

Table 2 shows that between 2008 and 2010, an estimated total of 559,528 VMMCs for HIV prevention have been done in the 13 priority countries, with a large majority (417,974) done in 2010. Kenya has carried out the largest number (232,287) of VMMCs, followed by South Africa (145,475) and then Zambia (81,849). Table 2 also shows the classification of countries according to DOI adopter status. The innovator (8% of countries), Kenya, started performing VMMCs in or before 2008 and reached more than 10,000 VMMCs in 2008. Early adopters (23% of countries), South Africa, Zambia, and Swaziland, started in or before 2008. The early majority (38% of countries), Botswana, Zimbabwe, Tanzania, Namibia, and Mozambique started in 2009. The late majority (15% of countries), Uganda and Rwanda, started in 2010 and did more than 1,000 VMMCs. The laggards (15% of countries), Malawi and Lesotho, did fewer than 1,000 VMMCs in 2010. The distribution of the total scale-up scores reflecting adoption status among the 13 countries in this study compares well with the distribution proposed by the DOI theory. Also highlighted in Table 2, only Kenya appears to be on track towards achievement of the DMPPT-estimated 80% coverage goal by 2015, having already achieved 61.5% of the DMPPT target. None of the other countries—including the early adopters—appear to be on track to achieve their targets.

**Table 2:** Service delivery statistics.

Countries	Number of Male Circumcisions Done in Each Calendar Year				DOI Adopter Status Classification	Estimated Number of VMMCs to Reach 80% Coverage	Achievement towards 80% Coverage
	2008	2009	2010	Total			
Kenya^a^	11,663	80,719	139,905	232,287	Innovator	377,788	61.5%
South Africa	5,190	9,168	131,117	145,475	Early adopter	4,333,134	3.4%
Zambia	2,758	17,180	61,911	81,849	Early adopter	1,949,292	4.2%
Swaziland	1,110	4,336	18,869	24,315	Early adopter	183,450	13.3%
Botswana	0	5,424	5,773	11,197	Early majority	345,244	3.2%
Zimbabwe	0	2,801	11,176	13,977	Early majority	1,912,595	0.7%
Tanzania	0	881	28,562	29,443	Early majority	1,373,271	2.1%
Namibia	0	224	1,763	1,987	Early majority	330,218	0.6%
Mozambique	0	100	7,633	7,733	Early majority	1,059,104	0.73
Uganda	0	0	9,052	9,052	Late majority	4,245,184	0.2%
Rwanda	0	0	1,694	1,694	Late majority	1,746,052	0.1%
Malawi	0	0	300	300	Laggard	2,101,566	<0.1%
Lesotho	0	0	219	219	Laggard	376,795	0.1%
**Total**	**20,721**	**120,833**	**417,974**	**559,528**		**20,333,693**	**2.68%**

These data were compiled by the PEPFAR Male Circumcision Technical Working Group and largely reflect data collated from sites funded by this agency.

a Nyanza Province only.

Table 3 shows the scoring scheme for each of the six key elements derived from the WHO/UNAIDS operational guidance, reflecting also the time when the different milestones were achieved (earlier completion resulting in higher scores). Table 4 shows the scores for each element by country and reflects the progress in scaling up key program elements in all of the priority countries. The total scale-up scores calculated for each of the 13 countries ranged from a low of 4 (Mozambique) to a high of 17 (Kenya) out of a maximum score of 18. To date, all countries have conducted a situation analysis to assess the acceptability of introducing VMMC programs with the support of a WHO toolkit [27]. At least seven countries had conducted their situation analyses in 2008, within a year of the release of the WHO/UNAIDS recommendations. Beyond the conduct of the situation analysis, progress towards the other key elements of scale-up varies significantly among the 13 countries.

**Table 3:** VMMC key elements of program scale-up scoring key.

Score	Situational Analysis Completed (Full or Selective)	Leadership: Prominent National Champion Engaged	Leadership: National Dedicated Focal Point in Place	VMMC Policy or Similar Guidance Approved	National Strategy and Operational/Implementation Plan Approved	Pilot/Demonstration Sites: Government Involvement
3	Completed all or some elements before the end of 2008	Influential national leader/advocate engaged for VMMC in 2007	National VMMC task force constituted that meets regularly by end of 2008	Formal policy or guidance, either separate or integrated into other national policy, approved by end 2008	Approved by end of 2008	Pilots set up with government engagement by 2008
2	Completed all or some elements before the end of 2009	Influential national leader/advocate engaged for VMMC in 2009	National VMMC task force constituted by end of 2009	Policy or equivalent approved during 2009	Approved during 2009	Pilots set up with government engagement by 2009
1	Completed all or some elements before the end of 2010	Influential national leader/advocate engaged for VMMC in 2010	National VMMC task force constituted by end of 2010	Draft policy, not yet approved or completed, during 2010	Draft or approved during 2010	Pilots set up with government engagement by 2010
0	None, or initial steps in progress	No leader/advocate engaged in VMMC early in the process	No national VMMC task force established	No policy or policy guidance	None	No government involvement or no pilot programs

In particular, large variation is seen in the leadership scores, with only Botswana, Kenya, Rwanda, and Swaziland having clearly identifiable prominent national champions for VMMC. We found that although almost all countries had a dedicated national focal person for VMMC in place, Botswana and Kenya had identified theirs within the first year of scale-up. Twelve out of the 13 countries had nationally approved policies for VMMC scale-up by the end of 2010, with Botswana and Kenya having their policies in place within a year of the WHO/UNAIDS recommendations. By the end of 2010, only Mozambique and Uganda did not have nationally approved scale-up strategies.

**Figure 3:**
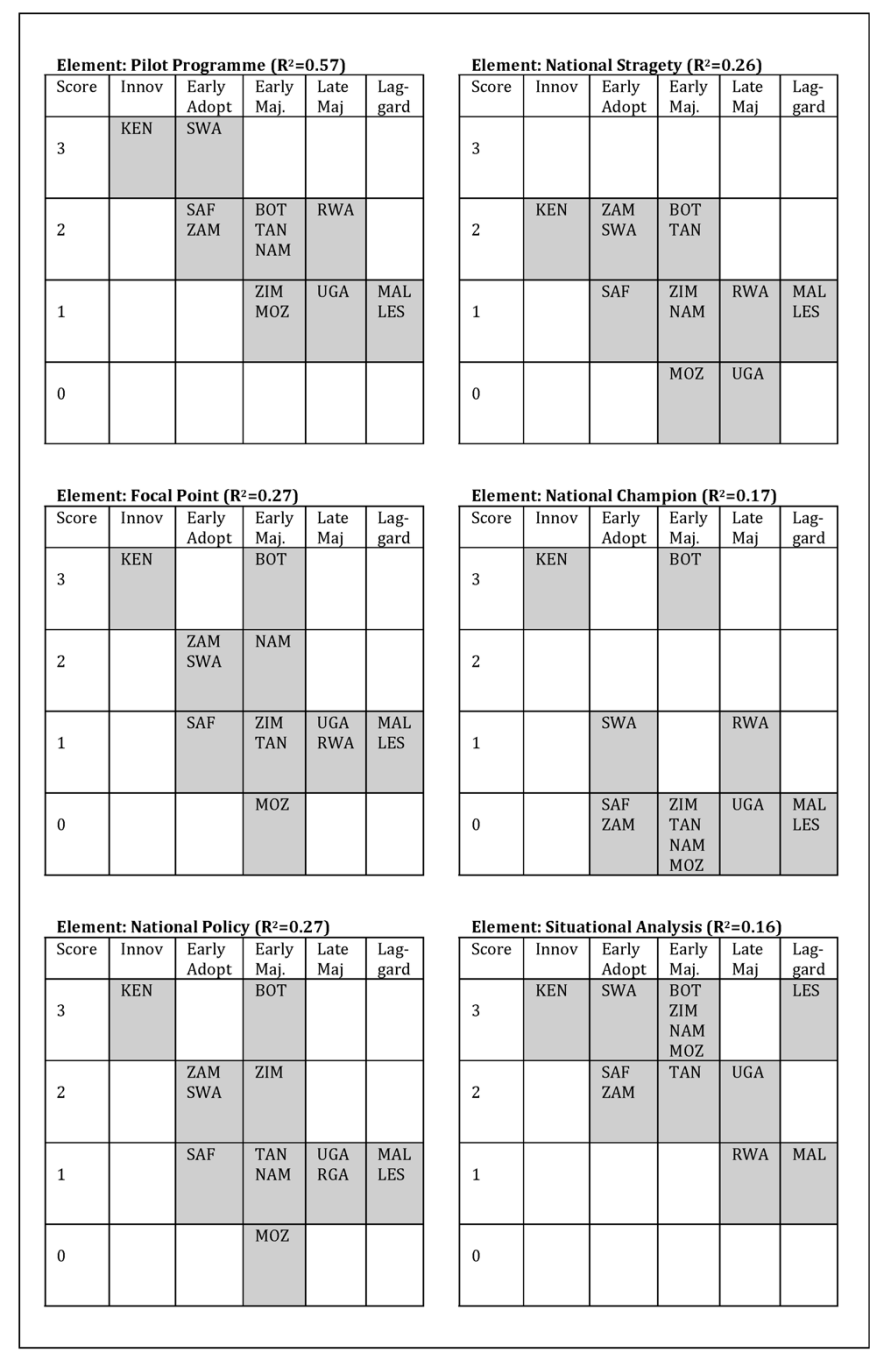
Association of scale-up element scores and Diffusion of Innovation adoption status. The correlation between each of the six elements of scale-up and DOI adoption status is shown in this figure. The scores obtained (ranging from 0 to 3) for each element by each country is shown (on the vertical axis) in relation to the adoption status (shown on the horizontal axis). Having a pilot program appears to be the strongest predictor of adoption status; this can be seen in the linear clustering of the countries. Conversely, having conducted a situational analysis appears to the least predictive of adoption status; the clustering of the countries is less linear and appears more random. BOT, Botswana; KEN, Kenya; LES, Lesotho; MAL, Malawi; MOZ, Mozambique; NAM, Namibia; RWA, Rwanda; SAF, South Africa; SWA, Swaziland; TAN, Tanzania; UGA, Uganda; ZAM, Zambia; ZIM, Zimbabwe.

Figure 3 summarizes the results of the R-squared analysis for each of the six key elements of scale-up identified in this study. The figure shows each of the 13 countries according to their DOI classification and their scores on the individual elements of VMMC program scale-up (Table 4), together with a crude estimate of association (R-squared) computed by assigning a linear score to the DOI classes. The association values can range from 0 to 1, with an R-squared value of 1 indicating perfect association. For this analysis, the association values ranged from 0.16 to 0.57. As the results suggest, conducting a pilot program (R-squared  =  0.57) may be the most important predictor of DOI class, followed by establishing a male circumcision focal point (R-squared  =  0.27), developing a national male circumcision policy (R-squared  =  0.27), and developing a national implementation strategy (R-squared  =  0.26). The role of having a national champion (R-squared  =  0.17) and conducting a situation analysis (R-squared  =  0.16) was shown to have a weaker predictive value on the likelihood of VMMC adoption and program scale-up.

**Table 4:** Country progress with scaling- up VMMC programs in focal countries (December 2010).

Country	Key Elements of VMMC Program Scale-Up						Total Scale-Up Score)
	Situational Analysis Completed^a^ (Full or Selective)	Leadership: Prominent National Champion Engaged	Leadership: National Dedicated Focal Point in Place	VMMC Policy or Similar Guidance Approved^a^	National Strategy and Operational/Implementation Plan Approved^a^	Pilot/Demonstration Sites: Government Involvement	
Botswana	3	3	3	3	2	2	16
Kenya	3	3	3	3	2	3	17
Lesotho	3	0	1	1	1	1	7
Malawi	1	0	1	1	1	1	5
Mozambique	3	0	0	0	0	1	4
Namibia	3	0	2	1	1	2	9
Rwanda	1	1	1	1	1	2	7
South Africa	2	0	1	1	1	2	7
Swaziland	3	1	2	2	2	3	13
Tanzania	2	0	1	1	2	2	8
Uganda	2	0	1	1	0	1	5
Zambia	2	0	2	2	2	2	10
Zimbabwe	3	0	1	2	1	1	8

Score range is 0 (lowest) to 3 (highest).

a The grading is based on the date of publication or official launch of these documents.

Specific examples of how the “user organization,” “resource team,” scaling-up strategy, and environment influenced scale-up are highlighted in Box 1. The type of policy document developed varies by country: some countries have stand-alone policy documents, while others have VMMC incorporated into other HIV prevention policies. Governments have been engaged in the setting up of pilot sites to test the feasibility of scale-up in all countries.

**Box 1. ExpandNet VMMC Components**

**User organization: the organizations and programs adopting the innovation**

- National Ministries of Health in all 13 priority countries have taken ownership and are leading the roll out in most countries.
- All countries have coordination structures (VMMC task forces) that are functioning to varying degrees. These task forces are partnerships between the Ministries of Health and the implementing partners. Kenya and South Africa also have provincial task forces.
- Human resource constraints—lack of personnel at national and facility level.

**Environment: the social, cultural, political, and economic context within which scaling up takes place**

- Notable political champions in Botswana and Kenya. Political changes in Botswana and South Africa affected initial trajectories both negatively and positively.
- Gaining political support – it has been a process to get political buy-in in some countries
- Global and national advocacy has moved some “early adopter” countries; peer pressure is working to bring the “late majority” on board. Preparatory stakeholders meetings were held in 2006 in five countries (Lesotho, Kenya, Tanzania, Swaziland, and Zambia) before the release of the WHO/UNAIDS recommendations. Regional consultations were held and national stakeholders meetings held in all countries after the release of the recommendations.
- Cultural context: issue raised of conflation with female genital mutilation (which occurs in some parts of Kenya and Uganda). In Lesotho, Malawi, and South Africa, cultural issues have been a challenge—the role of traditional providers has caused much debate and tensions. Lack of traditional male circumcision in Swaziland facilitated adoption.
- Legal issues: few countries have laws governing practice of traditional circumcisers. Task shifting, while successful in Kenya, is a challenge in other countries.

**Resource team: those involved in the development and testing of the innovation and/or seeking to promote its wider use**

- Developing countries look to WHO to provide norms and standards, therefore the timely release of the WHO/UNAIDS recommendations provided guidance for national policy and strategy development.
- The recommendations address the sociocultural, gender, and human rights issues that countries need to consider as well as health service issues, and therefore provide a comprehensive framework for policy development.
- The UN provided tools and guidance for scale-up, including operations guidance, legal regulatory tool, clinical manual for practice, and training, quality assurance, monitoring, and evaluation.
- Funding from donor organizations was made available and coordinated to enhance capacity of Ministries of Health and provider organizations in country.
- Partners in countries available to provide technical support for scale-up.
- Coordinated international leadership and advocacy supported country action.
- Strong partnerships between governments and non-governmental organizations have facilitated program scale-up in Kenya and, recently, Tanzania.

**Scaling up strategy: the means by which the innovation is communicated, disseminated, transferred, or otherwise promoted**

- Policy development was diverse across countries with differing types of policy instruments, e.g., Botswana has no separate policy but strategy with policy elements; Zambia sent Information note to Cabinet; Kenya developed policy guidelines; dedicated policies were developed in Lesotho, Namibia, South Africa, Swaziland, Uganda, and Zimbabwe.
- Country strategies developed that include objectives, target population, numbers of men to be reached, costs, service delivery strategies, resource mobilization, monitoring, and evaluation.
- DMPPT used to estimate cost, impact, pace of scale-up, and to develop or revise strategies.
- Different scale-up strategies have influenced program implementation. Most countries have “catch-up” strategies to reach adult men—Kenya, Swaziland, Zimbabwe, Zambia—however, implementation varies; Kenya has gone ahead with focused campaigns to achieve numbers, while Botswana is focusing on integrated service delivery.
- Demand creation—matching services to demand is difficult.
- Communicating partial protection and risk compensation are challenging.

## Discussion

We found that the DOI theory was most useful in analyzing the adoption and initial implementation of VMMC programs, while the ExpandNet theory helped to explore the factors that have facilitated or hindered scale-up. The analysis shows that although progress in VMMC program implementation has been made in all 13 countries within the first 3 y of the release of the WHO/UNAIDS recommendations, much more needs to be done if countries are to take programs to scale and achieve their targets of circumcising 80% of eligible men by 2015. The innovator country, Kenya, and the early adopters (South Africa, Zambia, and Swaziland) initiated VMMC program implementation soon after the release of the recommendations by adopting national policies and strategies and starting to perform VMMCs as part of pilot programs in 2008. However, only Kenya appears to be on track to achieving 80% coverage by 2015. To date, VMMC programs in priority countries have reached only approximately 3% of the target coverage level of 80% of eligible African men proposed by Njeuhmeli et al. [22]. Clearly an accelerated pace of VMMC service delivery is needed to take programs to scale and to maximize the impact of the programs.

Although the rate of adoption of VMMC programs varies among the 13 priority countries, the initiation of program adoption and initial implementation as a whole have been carried out relatively quickly compared to other public health interventions. Although there is much more research and scientific discovery now than ever before, the uptake of innovations does not seem to be much faster than it was 100 y ago. A review of nine landmark clinical procedures suggested that, on average, it takes a minimum of 6.3 y for research evidence to reach reviews, papers, and text books. They estimated that a further 9.3-y transition period is needed to implement the evidence from scientific publications [28]. ExpandNet case studies of scaling up reproductive health interventions indicate that about 10 y is required to scale up from pilot testing to nationwide expansion [25],[29]. Whereas in the case of VMMC, within 4 mo of the results of the three RCTs being released (in December 2006), WHO/UNAIDS had endorsed VMMC as a safe and effective means of HIV prevention and published recommendations for the implementation and scale-up of VMMC programs [19]. Less than a year later, pilot programs were initiated in the priority countries. Within 3 y of the release of the WHO/UNAIDS recommendations, the majority of the 13 priority countries had established national policies/strategies for HIV prevention that included VMMC and had begun to initiate service delivery. However, although the uptake of VMMC RCT evidence was relatively quick, it took almost two decades from the first cohort data [30] to the RCT meta-analysis, and implementation research is still insufficient.

Potential predictors of innovation and early adoption of the VMMC programs identified by this analysis include having a VMMC focal person, establishing a national policy, and having an operational strategy, as well as having a pilot or demonstration site with government involvement. These are important elements in confirming country ownership of the program. To create an environment for increasing country ownership and country-to-country learning, preparatory multi-stakeholder meetings were held in Kenya, Lesotho, Swaziland, Tanzania, and Zambia in 2006 [31]–[36]. Apart from Lesotho, all the other countries that held early stakeholder consultations are classified within the early majority, possibly also highlighting the significant role of the environment.

The DOI and the ExpandNet frameworks both postulate that the sociopolitical context and cultural relevance of an innovation are also critical factors influencing the widespread adoption of an innovation. The DOI theory also emphasizes that opinion leaders directly affect the adoption of an innovation; this explains why some innovations are quickly adopted in one setting but fail to take off in others. It is therefore interesting to note that from our analysis, the role of having a national champion engaged early on in the process does not appear to be a predictor of adoption status. However, despite the low association scores that having a national champion was shown to have in this analysis, we also know from our program implementation experience that former Botswana President Festus Mogae and Kenya's Prime Minister Raila Odinga were prominent leaders that championed VMMC programs in their respective countries [37]. In addition, in 2009, South Africa acquired a government that prioritized the strengthening of HIV programs, and on World AIDS Day in December 2009, the new president of South Africa publicly announced the government's determination to move the AIDS agenda forward, saying, “Let the politicization and endless debates about HIV and AIDS stop” and, regarding the need to struggle against AIDS as they had done with apartheid, “We have no choice but to deploy every effort, mobilize every resource, and utilize every skill our nation possesses.” [38],[39] This commitment at the highest level undoubtedly contributed to the significant number of VMMCs (131,117) performed in South Africa in 2010. Botswana had strong political support from their former President Festus Mogae; however, his presidency ended at the end of March 2008. This may explain why Bostwana has reached only 3.2% of its 2015 DMPPT target despite strong momentum in the initial phases.

In Kenya, consistent political support and ongoing community consultation have allowed implementation challenges to be addressed as they arise [40]. Kenya established a national VMMC task force that engaged all the key partners and was replicated at the provincial level. In addition, in 2007, Kenya initiated community mobilization activities that engaged community leaders and other key stakeholders in VMMC program implementation and generated the demand for services. These activities helped to overcome some of the initial political and cultural tensions and to accelerate service delivery [41] This further highlights the importance of the sociopolitical context and environment as well as sustained leadership at all levels (described in the ExpandNet framework) for moving `programs from initial adoption to scale-up. As suggested by the results of this study, it might therefore be that the consistency of political support and leadership, at all levels, is more important than just the initial engagement. This is due to the fact that while adoption is a one-time event, the scaling up of an innovation such as VMMC is an iterative process carried out over an extended period of time that requires continued political support and, in many instances, the input of decision makers to resolve implementation challenges as they arise. This ongoing need for political support and the involvement of decision makers is highlighted in the Kenya experience. An evaluation of the first year of VMMC program implementation in Kenya provides insights into some of the challenges of the Kenya program, including human resource constraints, inadequate infrastructure, and shortages of equipment and supplies, as well as difficulties with data management [40]. Despite challenges, the innovative responses that the Kenya Ministry of Health implemented in collaboration with other partners facilitated the translation of the national policies and programs into service delivery. Program managers facing shortages in human resources and inadequate infrastructure would not, without strong political support, be able to address these implementation challenges to the scale-up of an innovation.

While this analysis has not focused on the differences between country strategies for VMMC scale-up, the choice of strategy undoubtedly had an impact on VMMC program implementation and subsequent scale-up. For example, the scale-up strategy of Botswana has differed from that of other countries that acknowledge the need for a phase of vertical programs—the “catch-up” phase—to rapidly expand access to safe VMMC services in addition to a strategy for integration [19]. The Botswana strategy has focused on the integration of VMMC within existing health services; this is perhaps a reflection of the country's experience with scaling up ART. This may explain why Botswana has not performed as many VMMCs as the early adopter countries even though it had a VMMC focal person, policy, and strategy early on in the process. The timing of when countries conducted a situation analysis does not appear to have a bearing on the process of adoption of VMMC programs. However, for this analysis we did not take into account the type of situation analysis that was conducted or the process for dissemination and utilization of the situation analysis findings.

This analysis is not intended to criticize progress in any particular country; rather, it is an attempt to identify elements critical to success and underscore some of the challenges to scale-up. While medical male circumcision has many of the “CORRECT” characteristics, it is difficult to scale up the intervention, particularly as a result of human resources limitations in terms of both quantity and quality [42]. Also challenging are logistics and supply management: successful scale-up will in part depend on the definition and accessibility of commodities essential for VMMC programming and the appropriate allocation of resources to support commodity procurement and supply chain logistics [43].

This study suggests that the adoption of a health services innovation—the development of national policies and strategies, and the initiation of pilot programs—and program scale-up are distinct processes. This is clearly illustrated by the limited progress made towards scale-up by the early adopter countries. Thus, although the DOI theory was useful for predicting the adoption of VMMC as an innovation for HIV prevention, it was less useful as a model to describe what is actually needed to scale up VMMC services. The ExpandNet framework helps to provide some insights into the critical components required for scale-up.

### Limitations

The VMMC scale-up process has not been well documented in countries and therefore data were obtained from limited sources. The total scale-up score has not been used or validated in other programs and is reliant on assessments by a number of key informants who have been closely involved in VMMC scale-up at global and national levels. These individuals were interviewed and the information triangulated in an attempt to limit the potential bias. We limited our predictors of adoption status to the elements defined in the WHO/UNAIDS operational guidance, and yet there may be other important program elements that are not well captured in the guidance. We have attempted to draw out some of these other factors in the discussion but recognize that there may be other elements of program adoption that are not easily quantifiable.

Although the scores have captured the time when the different milestones were recorded and published, these only indirectly reflect the timing and pace of scale-up. The date of publication may have been several months after the work was initiated and/or completed, so the impact of the element may have been realized. The number of male circumcisions performed in priority countries may be underestimated as the data largely reflects male circumcisions done through programs funded by the United States President's Emergency Plan for AIDS Relief (PEPFAR). However, in many of the priority countries, initial male circumcision sites have been set up by PEPFAR implementing partners working closely with government. Finally, the quantitative analyses included in this study were based on a total sample of 13 countries. As such, although the results are indicative of experiences of these specific countries, the ability to generalize these findings to other contexts is limited.

### Conclusion

Three years after the WHO/UNAIDS recommendations to expand, promote, and integrate VMMC into comprehensive HIV prevention packages, VMMC has been adopted as a national HIV prevention strategy and implementation has been initiated in all of the priority countries. Policies, national scale-up strategies, and pilot projects have been put in place, and by the end of 2010, approximately 559,528 VMMCs had been performed in the priority countries, and yet this represents only about 3% of what is needed to achieve country-derived targets. The variability in progress in scale-up of male circumcision is evident, and the two diffusion frameworks suggest that the adoption of VMMC as a HIV prevention innovation does not guarantee scale-up. A key lesson is the importance of not only being ready to adopt a new intervention but also ensuring that those factors that accelerate and sustain program implementation are built and maintained. The most successful national program exhibited country ownership and sustained leadership at all levels, in addition to the adoption of a national policy and strategy to translate the research into a viable program.

## Abbreviations

DMPPT: Decision Makers' Program Planning Tool
DOI: Diffusion of Innovations
PEPFAR: United States President's Emergency Plan for AIDS Relief
RCT: randomized controlled trial
UNAIDS: Joint United Nations Programme on HIV/AIDS
VMMC: voluntary medical male circumcision
WHO: World Health Organization
